# Health-Related Quality of Life in Patients With Type 2 Diabetes Mellitus

**DOI:** 10.7759/cureus.88359

**Published:** 2025-07-20

**Authors:** Faisal Wali Ahmed, Junaid Ayaz Khan, Sunaira Hayat, Hafsa Ahmed, Mehryab Jawaid, Ayesha Haider, Malik Abdullah Rasheed, Hamna Khursheed, Fariha Altaf, Fahad A Khan, Muhammad Hamza

**Affiliations:** 1 Critical Care Medicine, King Saud Medical City, Riyadh, SAU; 2 Internal Medicine, Sun Yat-Sen Medical College, Guangzhou, CHN; 3 Medicine, Dow University of Health Sciences, Karachi, PAK; 4 Internal Medicine, Mayo Hospital Lahore, Lahore, PAK; 5 Medicine, Combined Military Hospital, Multan, PAK; 6 Medicine, King Edward Medical University, Lahore, PAK; 7 Internal Medicine, Services Hospital Lahore, Lahore, PAK; 8 Medicine, Multan Medical and Dental College, Hasilpur, PAK; 9 Obstetrics and Gynaecology, Tameside and Glossop Integrated Care NHS Foundation Trust, Manchester, GBR; 10 Medicine, Combined Military Hospital Multan, Multan, PAK; 11 Medicine, MedNova Research Institute, Islamabad, PAK

**Keywords:** chronic illness, diabetes complications, health-related quality of life, qol-q diabetes, sf-36, treatment type, type 2 diabetes mellitus

## Abstract

Background: People with type 2 diabetes mellitus (T2DM) can have both physical and mental problems that tend to lower their health-related quality of life (HRQoL). While T2DM is a serious issue for health worldwide, not much is known about its influence on the quality of life (QoL) for patients in Pakistan. The main goal of this study is to measure the QoL in patients with T2DM in Pakistan and to see how a range of factors can impact their HRQoL.

Methods: The research involved completing a cross-sectional study of 385 patients with T2DM from outpatient and diabetes care centers in Pakistan. Information was gathered using both the Diabetes Quality of Life Questionnaire (QoL-Q Diabetes) and the Short Form-36 Health Survey (SF-36). Both tests, i.e., Kolmogorov-Smirnov and Shapiro-Wilk, were applied to check if the data followed the usual pattern. It also used descriptive statistics, Pearson correlation, independent samples t-tests, one-way ANOVA, chi-square tests, and linear regression methods. We used IBM SPSS Statistics version 26, along with a p-value lower than 0.05, when carrying out statistical analysis. The interview process took place from November 2024 to April 2025.

Results: The analysis pointed out that scores for HRQoL varied significantly depending on the period of the disease, the care offered, and whether there were complications. On the Pearson correlation, a small and noticeable relationship (r = 0.074, p < 0.001) was found between the QoL-Q Diabetes and SF-36 scores. Those who lived with diabetes and treated it with insulin for a long time had scores that were somewhat lower for HRQoL. Although several differences between groups were statistically significant, their effect sizes were small, suggesting limited clinical relevance. The gaps that were noted in the study were mostly small in real-life situations. People who suffer from additional health problems, such as nephropathy and neuropathy, live with a noticeably lower HRQoL.

Conclusion: In conclusion, our research shows that T2DM has a moderate influence on patients' HRQoL in Pakistan. The most important factors influencing this are the type of treatment, the duration of T2DM, and any complications that develop. Although some group differences were statistically significant, the effect sizes were small, indicating minimal clinical impact. Therefore, individualized and comprehensive care remains essential to improve patients' overall QoL meaningfully.

## Introduction

Type 2 diabetes mellitus (T2DM) is characterized by insulin resistance and progressive β-cell dysfunction, leading to impaired glucose uptake and elevated blood glucose levels. Problems in insulin synthesis, release, and signaling, as well as effects from genes, the environment, and lifestyle, such as gut dysbiosis, aid the progression of the disease and cause related vascular complications [1,2]. During the later years of the 20th century, T2DM quickly grew into a worldwide epidemic, impacting wealthy countries first, but now affecting more in developing regions. Studies predict that T2DM is set to increase at a significant rate, mainly in the Middle East, Africa, and Asia, and China, where cases could be more than double by the year 2030 [3].

Obesity increases the chance of T2DM, but some people with a genetic risk may experience insulin resistance early, which leads to them becoming less healthy and gaining weight. These findings challenge the traditional understanding of obesity as the primary driver of T2DM and instead highlight insulin resistance as a critical precursor in its development [4]. An extensive study found that 5.4% of the population suffered from type 2 diabetes, and 54% of these cases were unknown to them. The older a person was, the more likely they were to have higher rates, as were females, those with higher body mass indices, and those living in rural areas [5].

Both nonalcoholic fatty liver disease (NAFLD) and T2DM are linked because each can raise the risk of the other. The main problem leading to these causes is insulin resistance, which means that integrated procedures for screening and treatment are very important [6]. Health, HRQoL, and QoL are sometimes used interchangeably in the studies. It should be understood that defining health-related quality of life (HRQoL) properly is to show how an individual’s health directly influences their total well-being or how they feel about their health [7]. Measuring QOL and HR-QOL in people who have type 2 diabetes allows us to see the differences between the two concepts. Although HR-QOL is concerned just with health, overall QOL includes various factors that boost a person's general well-being, covering social situations, moods, and the environment [8].

Nowadays, HRQoL is being increasingly recognized for measuring the health of people in a population and the workings of healthcare services. Nevertheless, because of these challenges, it is not always possible to compare demand for healthcare across regions with different socioeconomic levels [9]. HRQoL covers several different aspects and is usually measured using several indicators, so each indicator alone cannot fully show someone's overall health status. Having all these measures in one score gives a more transparent and more trustworthy way to understand a person's HRQoL [10].

Individuals with type 2 diabetes usually feel that their HRQoL is lower than average. However, it is found that younger patients, those who have had diabetes for a short time, have fewer issues, and well-controlled blood sugar levels tend to have better results. Seeing these factors makes it easier to manage diabetes and support patients [11].

It is essential to understand HRQoL in people with T2DM to create effective and patient-focused treatment approaches. It makes it easier for healthcare providers to note both medical and emotional challenges, therefore helping patients stick to treatments and being more satisfied. The aim is to examine how QoL measures in T2DM patients and the factors that govern this.

Rationale

Even though researchers around the world have studied how patients with T2DM feel about their health, studies from Pakistan remain few. Due to the country's social, economic, and healthcare challenges, the experience of patients might vary significantly from that of patients in more developed nations. The difficulty of obtaining proper medical care, facing high costs, not knowing much about diabetes, and trying to control multiple related conditions can decrease the QoL for people living with it in Pakistan. Although some investigations on HRQoL have included diabetic patients from specific areas, these studies usually fail to represent the whole country or region. In addition, because researchers use different methods and tools, their findings do not always match. Since T2DM is becoming more common in Pakistan, we require fresh and local information to understand how the disease affects patients day-to-day. The goal of this research is to close that gap by looking into the HRQoL of T2DM patients in Pakistan and figuring out which elements have the most significant effect on it. On this basis, suitable and personalized diabetes treatment methods can be used in Pakistan, and public health strategies can be updated.

Primary Objective

The primary objective of the study is to examine the HRQoL of patients with type 2 diabetes in Pakistan.

Secondary Objectives

Secondary aims are to determine age, gender, education, economic level, and other demographic features that could influence HRQoL and to investigate the impact of disease duration, type of diabetes, presence of complications, comorbidities, and treatment methods on HRQoL.

The research also explores how emotional health and help from others influence HRQoL.

## Materials and methods

Study design and methods

The study employed a cross-sectional design to investigate the QoL for individuals with type 2 diabetes. Data were collected over a four-month period from November 2024 to April 2025 from various outpatient clinics and diabetes care centers across Pakistan, including both government-managed and private institutions. As a result, the research included patients from many different ethnic, cultural, and income backgrounds.

Data collection was done by structured interviews or self-administered surveys, depending on what the participant preferred and could understand. The researchers were authorized to use specific tools to measure QoL and gather background information. During regular clinic visits, people were approached and were only included if they gave their consent willingly before any data was collected.

Using this research method, researchers were able to find out about the general QoL in patients with T2DM and discover challenges they face while managing their disease. The purpose of the findings is to give helpful directions for upgrading diabetes care and support systems in Pakistan.

Sample size and technique

The total number of T2DM cases among the general population is unknown; as a result, the population was treated as infinite for the study. It is calculated with this equation:

Z² x p (1-p) / d²= sample size

Z here is the standard value for the confidence needed, p is the expected proportion based on findings from earlier studies, and d is the allowable margin for error in the estimated results. The value of Z was set at 1.96, and at a 95% confidence level, the margin of error (d) was determined to be 0.05. Since a similar Pakistani study found that 50% of type 2 diabetes patients had lower health quality, the researchers chose p = 0.50 for maximum sample size [12].

The values chosen allowed for a sample size of 385 participants. To account for potential non-responses or missing data, the sample size was increased to 410 participants. Those who took part in the study were selected conveniently from outpatient clinics and diabetes centers, which made it possible to include people who met the requirements and could join at that time. To address missing data, listwise deletion was used, meaning participants with missing responses for any variable were excluded from the relevant analysis. The approach was adopted to minimise possible biases that might be introduced due to the use of imputation methods and to maintain the reliability of the data that were used in the analysis.

The inclusion and exclusion criteria for participant selection are summarized in Table 1.

**Table 1:** Inclusion criteria and exclusion criteria for study participants

Inclusion criteria	Exclusion criteria
Anyone who is 18 years old or older	People who have type 1 diabetes mellitus
Experienced type 2 diabetes mellitus for at least six months	Those who are affected seriously by cognitive impairment or psychiatric illness
Willingly taking part and signing the consent form	Those dealing with acute or end-stage conditions that involve challenges with their quality of life
Know how to speak in Urdu or English	Pregnant women diagnosed with gestational diabetes

Data collection tools

For data gathering, we designed a questionnaire comprising three sections: demographics, diabetes QoL, and general health QoL. Both the SF-36 and QoL-Q Diabetes were used in their original form in this study. To assist participants in understanding the questions, verbal help was provided when necessary, but no official translation or cultural adaptation was made for either scale. Both validated scales and demographic questions created by the researcher were included in the questionnaire to ensure a complete set of data.

Demographic Information

The first part of the study collected background information to find out if personal, social, and other factors influence the QoL. Factors such as age, gender, marital status, education, occupation, and the length of time the person had diabetes were all included. These factors were analyzed to determine if there was an association with how people felt about their health and to learn more about the population itself.

Diabetes Quality of Life Questionnaire (QoL-Q Diabetes)

For the second part, the researchers relied on the Diabetes Quality of Life Questionnaire (QoL-Q Diabetes) developed by Burroughs et al. in 2004. The tool was created to determine how people with diabetes feel in terms of disease impact, their treatment satisfaction, their social and emotional well-being, and anything that distresses them because of diabetes. The questionnaire relies on a Likert-type scale, where one means life quality is poor and five means an excellent QoL. Better QoL is indicated by having high scores. The reliability of the QoL-Q Diabetes has been widely demonstrated by alpha values ranging from 0.76 to 0.92 across various populations. However, the use of this tool in Pakistan requires consideration of the local socioeconomic and cultural factors. While the tool is widely used in diabetes populations globally, Pakistan's unique healthcare challenges and sociocultural context may influence how patients interpret and respond to questions, particularly regarding diabetes-related distress and treatment satisfaction. Since the QoL-Q Diabetes has not yet been validated explicitly for the Pakistani population, future studies may benefit from the cultural adaptation of this tool to better reflect local experiences, including healthcare access, treatment barriers, and the psychosocial aspects of living with diabetes in Pakistan. Official approval was granted to conduct this study with the questionnaire [13].

Short Form-36 Health Survey (SF-36)

To assess several parts of HRQoL, this study applied the SF-36 Health Survey (SFHS). In 1992, Ware and Sherbourne developed the survey, which evaluates eight properties: physical exercise, health limitations, pain intensity, general health perceptions, energy levels, social engagement, emotional well-being, and psychological well-being. The domain scores range from 0 to 100, and a high score indicates that people perceive their health in that domain as better. The SF-36 has shown strong reliability in chronic illness groups, with a Cronbach's alpha of 0.90, confirming its consistency and dependability across different populations, including the general public. However, the SF-36 is a general health questionnaire, and it may not comprehensively reflect the effects of diabetes-related complications, such as nephropathy or retinopathy, which are of particular interest to T2DM patients in Pakistan. Despite several studies using the SF-36 in South Asia, its administration in Pakistan should be taken with caution because cultural and contextual differences could contribute to changing the nature of how patients perceive and report issues relating to mental health, pain, and social functioning. This suggests that the results should be interpreted carefully, especially when applied to Pakistani T2DM patients to evaluate HRQoL. It was officially granted that the questionnaire could be used in the research. The original SF-36 survey remained unchanged. Appreciation is acknowledged for the formulation of SF-36 by the RAND Corporation. It is used in terms of RAND regarding the SF-36 tool [14,15].

Procedure

Participants were chosen from outpatient clinics and diabetes care centers only after they gave informed consent. Participants in the survey either answered the questions independently or participated in interviews conducted by trained data collectors. Personal details were removed from all the data to maintain confidentiality. In this way, the information collected was accurate, ethical, and respectful, and included participants from different backgrounds.

Statistical analysis

IBM SPSS Statistics for Windows, version 26.0 (IBM Corp., Armonk, NY) was used to analyze the data. Results with a p-value less than 0.05 were regarded as being statistically significant. The descriptive statistics, including means, standard deviations, frequencies, and percentages, were computed to summarise the participant demographics and HRQoL scores. The Kolmogorov-Smirnov and Shapiro-Wilk tests were used in determining the normality of the data. These tests are applied to determine whether or not the data are significantly different from a normal distribution. The analysis revealed that results on the SF-36 Health Survey were normally distributed (Kolmogorov-Smirnov = 0.200 and Shapiro-Wilk = 0.502, p-values). However, there were some inconsistencies in the QoL-Q Diabetes scores with Kolmogorov-Smirnov indicating non-normal distribution (p = 0.020) and Shapiro-Wilk indicating approximate normal distribution (p = 0.129). Since the sample size was quite large (n = 385) and Shapiro-Wilk is reliable even in large samples, parametric tests were considered reasonable in both data sets. Pearson correlation was applied to analyze the connection between overall HRQoL (SF-36) and diabetes-specific HRQoL (QoL-Q Diabetes). This relationship has a weak but statistically significant correlation coefficient (r = 0.074) and p < 0.001. The effect size was small, which has the implication that, although the relationship between the variables is statistically significant, the magnitude of their relation is not very strong. One-way ANOVA was conducted to ascertain the impact of the duration of diabetes and the mode of treatment on the HRQoL. Both diabetes duration (p = 0.005 in QoL-Q Diabetes, p = 0.002 in SF-36) and treatment type (p = 0.05 in QoL-Q Diabetes, p = 0.04 in SF-36) showed statistically significant variations.

Nevertheless, the effect sizes were small, with 0.004 and 0.011 representing the values of 0.2 in QoL-Q Diabetes and SF-36, implying that although these differences are statistically significant, clinical significance might be minimal. A linear regression was performed to investigate the possibility of predicting SF-36 outcomes using the scores in QoL-Q Diabetes. The regression analysis showed a statistically significant but weak relationship between them (0.074, p < 0.001). The relationship here was found to be statistically significant, but of minimal practical significance. The relationship between diabetes-related complications and types of treatment was evaluated with the help of Chi-square tests. Both treatment type and complications exhibited significant associations (p < 0.001 and p = 0.003, respectively), which show that patients who received more intense treatments (e.g., insulin therapy) tended to report more complications, including retinopathy and nephropathy.

Ethical considerations

The study was carried out following the required ethical guidelines for studies involving people. Before the study started, the protocol was studied and approved by the Mednova Research Institute, Islamabad’s Institutional Review Board (IRB-2024-0020). Because of this approval, it was guaranteed that ethical principles such as respect for persons, beneficence, and confidentiality were consistently observed.

Everyone taking part in the study was given thorough information about the reasons for it, the steps involved, any risks, and what benefits they might gain. Every participant gave informed consent in writing before being included in the study. No one was forced to take part in the survey, and people could drop out at any time without any trouble. Confidentiality was maintained throughout the study, with personal identifiers removed from all data to ensure privacy.

## Results

Table 2 shows that most participants were young adults aged 18-30 years (n = 259, 67%) and predominantly male (n = 252, 65%). Marital status distribution revealed that 145 (38%) were married and 116 (30%) were divorced. Concerning education, the largest group had secondary school education (n = 125, 32%), followed by those with high school education (n = 86, 22%) and primary school education (n = 86, 22%). The employment status is varied, with 102 (26%) self-employed individuals, 99 (26%) unemployed individuals, and 92 (24%) retirees. Most of them had type 2 diabetes, which lasted one to six years (n = 221, 58%). The most frequent treatments were insulin therapy (n = 144, 37%), oral hypoglycemics (n = 88, 23%), and combination therapy (n = 70, 18%). The most commonly reported complications include nephropathy (n = 104, 27%) and retinopathy (n = 85, 22%). In addition, 280 respondents (73%) reported the presence of a minimum of one other chronic illness.

**Table 2:** Demographic characteristic of the participants (N = 385) f = frequency, % = percentage

Variable	f	%
Age	-	-
18-30 years	259	67
31-40 years	89	23
41-50 years	27	7
51-60 years	10	3
Gender	-	-
Male	252	65
Female	133	35
Marital status	-	-
Single	85	22
Married	145	38
Divorced	116	30
Widowed	39	10
Educational level	-	-
No formal education	42	11
Primary school	86	22
Secondary school	125	32
High school diploma	86	22
Undergraduate	35	9
Postgraduate	11	3
Employment status	-	-
Student	39	10
Employed	53	14
Self-employed	102	26
Unemployed	99	26
Retired	92	24
Duration since diagnosis of type 2 diabetes	-	-
Less than one year	78	20
One to three years	110	29
Four to six years	111	29
Seven to 10 years	70	18
More than 10 years	16	4
Type of diabetes treatment	-	-
Diet control only	56	14
Oral hypoglycemic agents	88	23
Insulin therapy	144	37
Combination of oral medication and insulin	70	18
Other	27	7
Presence of diabetes-related complications	-	-
None	72	19
Retinopathy (eye problems)	85	22
Nephropathy (kidney problems)	104	27
Neuropathy (nerve damage)	65	17
Cardiovascular disease	39	10
Foot ulcers	20	5
Do you have any other chronic illnesses?	-	-
Yes	280	73
No	105	27

Table 3 indicates that the Kolmogorov-Smirnov and Shapiro-Wilk tests were used to test the normality of the data. In the case of the SF-36 Health Survey, both tests, Kolmogorov-Smirnov (p = 0.200) and Shapiro-Wilk (p = 0.502), demonstrated a normal distribution. In QoL-Q Diabetes, there were some inconsistent findings: Kolmogorov-Smirnov showed data non-normality (p = 0.020), but Shapiro-Wilk demonstrated that the sample followed an approximate normal distribution (p = 0.129). Based on the sample size (n = 385) and increased reliability of the Shapiro-Wilk test conducted in such samples, the necessity to use parametric tests was justified in both data sets. However, the QoL-Q Diabetes scores are assumed to be used with caution.

**Table 3:** Normality test to check equal distribution among the Quality of Life Questionnaire and Short-Form Health Survey df = degree of freedom; parametric test = p > 0.05; non-parametric test = p ≤ 0.05

Variables	Kolmogorov-Smirnov			Shapiro-Wilk		
	Statistic	df	p	Statistic	df	p
Quality of Life Questionnaire	0.050	385	0.020	0.994	385	0.129
Short-Form Health Survey	0.039	385	0.200	0.996	385	0.502

Table 4 highlights the links between the QoL-Q and the SFHS. We found a noticeable relationship between the QoL-Q and the SFHS, with a correlation value of 0.074. It points out that there is a modest relationship between the two variables. The correlation, as shown by the numbers, is not very powerful despite the positive relationship between the variables.

**Table 4:** Intercorrelations between study variable * = p < 0.05, ** = p < 0.001 considered significant; correlation = Pearson correlation

Variable	Quality of Life Questionnaire	Short-Form Health Survey
Quality of Life Questionnaire	-	0.074^**^
Short-Form Health Survey	0.074^**^	-

Table 5 contrasts the scores of the QoL-Q and the SFHS for participants who have chronic illnesses (Yes) with those who do not (No). For this survey, the average score for people with chronic illness is 124.03 ± 7.49, while for those without chronic illness is 124.12 ± 6.66. The test results tell us that there is a significant difference between the two groups (p < 0.001), but the size of the effect is very small (Cohen's D = 0.01). The mean score for people affected by chronic illness on the Short-Form Health Survey is 86.37, and for those not affected by chronic illness, it is 86.86. A significant difference was found using the t-test, but with a very small effect size (Cohen's D = 0.07). Both variables had significant differences between people with and without chronic illnesses. Nonetheless, the differences found are not significant enough to have much impact.

**Table 5:** Comparison among variables (chronic illness) M = mean, SD = standard deviation, LL = lower limit, UL = upper limit; Cl = confidence interval; independent t-test; ** = p < 0.001 considered significant

Variable	Yes (N = 280); M ± SD	No (N = 105); M ± S.D	t	P	Cl 95% LL	UL	Cohen’s D
Quality of Life Questionnaire	124.03 ± 7.49	124.12 ± 6.66	-0.114	<0.001^**^	-1.733	1.542	0.01
Short-Form Health Survey	86.37 ± 7.19	86.86 ± 7.73	-0.578	<0.001^**^	-2.137	1.166	0.07

Table 6 shows how the QoL-Q and the SFHS compare for people who have had type 2 diabetes for various lengths of time. The mean scores for the QoL-Q by duration group are less than one year (123.62 ± 7.05), between one and three years (123.59 ± 7.57), between four and six years (124.46 ± 7.37), between seven and 10 years (124.39 ± 7.32), and more than 10 years (125.13 ± 5.46). The p-value found in the one-way ANOVA test was 0.005, showing that the result is significant, and the F-ratio was 0.390 with a very small effect size (η2) of 0.004. For the Short-Form Health Survey, people in each duration group scored the following: less than one year (85.88 ± 7.57), one to three years (86.80 ± 7.77), four to six years (85.98 ± 6.58), seven to 10 years (86.89 ± 7.73), and more than 10 years (89.44 ± 6.11). The p-value shows that the difference between groups is statistically significant, and the results show an F-ratio of 1.012 and an effect size of 0.011. In short, there are statistically meaningful differences between periods after diagnosis using both the QoL-Q and the SFHS. While there are significant differences, their actual impact is fairly limited, suggesting very small effect sizes.

**Table 6:** Comparison of variables (duration since diagnosis of type 2 diabetes) M = mean, SD = standard deviation, F = ratio of variance between groups to within groups, η2 = effect size; one-way ANOVA; ** = p < 0.01 considered significant

Variable	Less than one year (N = 78); M ± SD	One to three years (N = 110); M ± SD	Four to six years (N = 111); M ± SD	Seven to 10 years (N = 70); M ± SD	More than 10 years (N = 16); M ± SD	p	F (4,380)	η2
Quality of Life Questionnaire	123.62±7.05	123.59±7.57	124.46±7.37	124.39±7.32	125.13±5.46	0.005^**^	0.390	0.004
Short-Form Health Survey	85.88±7.57	86.80±7.77	85.98±6.58	86.89±7.73	89.44±6.11	0.002^**^	1.012	0.011

Table 7 compares the QoL-Q and SF-36 scores across different types of treatment in diabetes patients. For the QoL-Q, the diet control group had the highest mean score (124.64 ± 7.46), followed by oral hypoglycemic drugs (123.60 ± 6.18), insulin therapy (123.82 ± 7.85), combination therapy (124.33 ± 7.70), and other treatments (124.85 ± 5.99). Despite these differences in means, the p-value was 0.05, and the effect size (η² = 0.003) was tiny, indicating that the practical significance of these differences is minimal. For the SF-36, the mean scores were as follows: diet control (85.79 ± 7.95), oral hypoglycemic drugs (85.44 ± 6.89), insulin therapy (86.35 ± 6.76), combination therapy (88.93 ± 8.04), and other treatments (85.96 ± 7.54). The p-value for SF-36 was 0.04, suggesting statistically significant differences between the treatment groups, and the effect size (η² = 0.027) indicates a small but somewhat more meaningful impact in comparison to the QoL-Q. In conclusion, while both the QoL-Q and SF-36 show significant differences between treatment groups, the SF-36 appears to have a somewhat greater practical influence on HRQoL. However, the effect sizes for both measures are small, indicating that while treatment type does influence HRQoL, the impact is modest.

**Table 7:** Comparison of variables (type of diabetes treatment) M = mean, SD = standard deviation, F = ratio of variance between groups to within groups, η2 = effect size; one-way ANOVA; * = p < 0.01 considered significant

Variable	Diet control only (N = 56); M ± SD	Oral hypoglycemic agents (N = 88); M ± SD	Insulin therapy (N = 144); M ± SD	Combination of oral medication and insulin (N = 70); M ± SD	Other (N = 27); M ± SD	p	F (4,380)	η2
Quality of Life Questionnaire	124.64±7.46	123.60±6.18	123.82±7.85	124.33±7.70	124.85±5.99	0.05^*^	0.318	0.003
Short-Form Health Survey	85.79±7.95	85.44±6.89	86.35±6.76	88.93±8.04	85.96±7.54	0.04^*^	2.601	0.027

Table 8 presents the findings from the linear regression analysis involving the scores from the QoL-Q and SF-36 Health Survey. For the Constant in the regression model, B = 77.188, SESESE (B) = 6.390, and the p-value is far below 0.001, indicating that it is significant in the model. The QoL-Q shows a coefficient (B) of 0.075 with confidence in its value falling within -0.026 and 0.176. Since β is only 0.074, it is clear that there is a small and positive link between the independent and dependent variables. The fact that this variable's value is less than 0.001 means that the relationship is noticeable. All in all, it was found that the QoL-Q is related to the dependent variable through regression analysis. In addition, since β came out at 0.074, it suggests that the effect is not significant.

**Table 8:** Linear regression for the Quality of Life Questionnaire and Short-Form Health Survey B = coefficient, SE = standard error, β = standardized coefficient, LL = lower limit, UL = upper limit; Cl = confidence interval, ** = p < 0.01 considered significant

Variable	B	95% Cl		SE	β	P
		LL	UL			
Constant	77.188	64.624	89.752	6.390	-	<0.001^**^
Quality of Life Questionnaire	0.075	-0.026	0.176	0.051	0.074	<0.001^**^

Table 9 demonstrates the prevalence of diabetes-related complications in connection with the length of time since the diagnosis of type 2 diabetes and a specific type of diabetes treatment. The length of diabetes and the existence of complications were also connected in a statistically significant manner (df = 20, p = 0.003, 22.0), which implies that retinopathy, nephropathy, neuropathy, cardiovascular disease, and foot ulcers were observed more among patients with a longer diabetes disease experience, especially during the first four years of disease development. Similarly, there was a statistically significant correlation between the type of treatment of diabetes and the condition of complication (df =20, p < 0.001, x2 =18.6); the highest rate of complication was in patients under the treatment of insulin or a combination of an oral hypoglycemic agent with insulin. This indicates that people who receive higher intensities of treatments might be on higher levels or duration of diabetes.

**Table 9:** Descriptive statistics of demographic variables (presence of diabetes-related complications, duration since diagnosis of type 2 diabetes, and type of diabetes treatment) f = frequency; % = percentage; x2 = effect size; p = level of significance; p-values calculated using the chi-square test; the significance level is set at p < 0.05; ** = p < 0.01 considered significant

Variables	f	Less than one year	Duration since diagnosis of type 2 diabetes: one to three years	Four to six years	Seven to 10 years	More than 10 years	df	p	x^2^	Diet control only	Oral hypoglycemic agents	Type of diabetes treatment: insulin therapy	Combination of oral medication and insulin	Other	df	p	x^2^
Presence of diabetes-related complications	-	-	-	-	-	-	20	0.003**	22.0	-	-	-	-	-	20	<0.001**	18.6
None	72	17	23	14	14	4	-	-	-	13	10	31	13	5	-	-	-
Retinopathy (eye problems)	85	23	25	24	11	2	-	-	-	9	19	41	14	2	-	-	-
Nephropathy (kidney problems)	104	14	32	34	20	4	-	-	-	14	25	35	20	10	-	-	-
Neuropathy (nerve damage)	65	13	21	18	10	3	-	-	-	8	19	21	12	5	-	-	-
Cardiovascular disease	39	7	4	16	11	1	-	-	-	9	11	9	7	3	-	-	-
Foot ulcers	20	4	5	5	4	2	-	-	-	3	4	7	4	2	-	-	-

## Discussion

The study aimed to see how type 2 diabetes affects the HRQoL in Pakistan and to look at how factors such as age and gender can influence these results. We found that while the two tools, the QoL-Q and SFHS, yielded the same results, the relationship was meaningful but not particularly powerful. These results suggest that there is a need for more precise explanations of the differences between health, HRQoL, and QoL [7].

We found that chronic illness affects QoL only marginally, agreeing with studies showing that relationships and other meaningful activities are vital to happiness. It points out that the true QoL depends on health and different crucial aspects of life [16]. Our study showed that the QoL for people with and without chronic illness was very similar. This result fits with past studies, showing that both health status and social and developmental factors affect HRQoL and should be considered in any QoL assessment [17].

Our observations about small but meaningful changes in QoL over varying diabetes durations are in line with what is seen in existing research, which describes the harmful effects of complications and related illnesses on QoL; disease length alone appears to have little impact. This points out that taking care of diabetes-related conditions helps improve one's QoL [18]. We found that people’s QoL regarding their health decreased slightly as the disease progressed. It agrees with past studies that, when it comes to QoL, other health issues and depression generally play a bigger role than the length of diabetes. That's why it is essential to diagnose and treat complications and mental health problems as soon as possible in patients with type 2 diabetes to increase their QoL [19].

We observed that patients who controlled diabetes with healthy eating had a slightly higher level of QoL than those who relied on insulin. Likewise, studies have previously shown that good lifestyle habits and regular glucose checking help QoL, while illnesses and an extended period with the disease do the opposite [20]. We found that patients who took both insulin and combination therapy had slightly improved scores in QoL. This agrees with past studies, in which insulin therapy was found to help type 2 diabetes patients manage their health better and stick to their treatment plan [21].

We found that the link between the QOL-Q and SFHS is not strong, which could be due to similarities between health status and QoL, as mentioned in earlier studies that raise questions about the accuracy and stability of measuring HRQoL [7].

In the past, studies have demonstrated that the length of diabetes is closely related to a higher risk of both kinds of complications: microvascular and macrovascular. Our analysis revealed that patients who have had diabetes for a longer time are more at risk of developing nephropathy, retinopathy, and neuropathy. These outcomes stress that diabetes gets worse over time, and proper early management can slow the development of complications [22]. Our study supports findings that reveal complications increase as diabetes continues and when people use high-intensity treatments such as insulin. Therefore, personalized care is necessary to stop the disease from getting worse [23].

Limitations

This study has a few significant limitations that cannot be ignored. Initially, the cross-sectional method does not allow us to establish a direct link between clinical or demographic characteristics and HRQoL. Because data were taken just once, the development of diabetes management and its lasting effects on living quality could not be measured. Next, those participating were chosen easily from outpatient clinics and diabetes centers, and this approach might not cover a wide range of diabetic people in Pakistan. As a result, the findings may not apply to all people. Third, this study depended on answers from people, and there is a risk of biased responses, especially about their previous medical events, trouble during birth, and how they feel. Even though older people suffer from many diseases, there were very few of them included in the study sample. Since the findings depend on information from participants, the absence of actual tests or diagnoses could lead to both over- and underestimations of complications and chronic diseases.

Furthermore, although both the SF-36 and QoL-Q Diabetes represent reliable and well-established tools, their relevance in the Pakistani setting needs to be addressed. Pakistan interpersonal and health variations can interfere with the patient's answers to such questionnaires, especially on such aspects as the distress caused by diabetes and social support. These instruments were not directly tested on Pakistani samples, and thus, the findings should be considered with caution, given that localized differences in perception and response may have an impact on the results. Lastly, another major limitation is that no theoretical framework was provided to facilitate an analysis of HRQoL. Applying a theoretical focus, such as the stress-coping model or social support theories, could have been used, allowing for a more in-depth explanation of the interaction between psychosocial factors and diabetes-related distress and HRQoL. The absence of such a framework imposes constraints on the conceptual richness of the analysis and can be an obstacle to a more nuanced interpretation of the findings.

Future directions

Studies in the future should look at longitudinal data to track any changes in HRQoL as the disease develops, patients receive different treatments, and they change their lifestyle. This would help reveal clear connections between events and reveal ongoing changes over time. Adding measurements such as levels of HbA1c, body mass index, and lipids will boost the reliability of results and help in identifying stronger ties between health status and HRQoL. Having a larger sample of older people would be helpful, as diabetes is more likely to affect them with behavioral and emotional issues. In addition, studies using interviews or focus groups may provide a more comprehensive picture of patients’ experiences, beliefs, and coping mechanisms, thereby adding value to the results from research that relies on numerical data. Future studies should assess how initiatives such as community classes, group support models, and counseling for mental health benefit people with type 2 diabetes. In the future, researchers should consider adapting and validating both the QoL-Q Diabetes and SF-36 questionnaires to make them culturally relevant and comprehensively precise for the Pakistani population. This may entail administering focus groups or qualitative interviews to gain a closer insight into how patients in various parts of Pakistan receive and react to the items through such tools. These tools would be validated using psychometric testing in the Pakistani context, as it would ensure they capture the exclusive experiences and health issues of T2DM patients in the area adequately. Also, the theoretical framework should be considered in future research to perform the analysis of HRQoL. The psychosocial factors that would affect the HRQoL of the patients would have been better understood using the stress-coping model, the social support theories, or any other practical frameworks. A theoretical framework would be beneficial for putting the findings into perspective and informing interventions aimed at enhancing diabetes care and support systems in Pakistan.

## Conclusions

The findings of the current research indicate that the QoL among patients with type 2 diabetes in Pakistan is mostly moderate. It is influenced by factors such as the type of treatment, the duration of the disease, and the presence of complications. While HRQoL varied across different groups, the differences were relatively small. The results indicate that early diagnosis, personalized treatment, and comprehensive care, particularly psychological and social support, play a critical role in enhancing the QoL of such patients. Addressing both medical and social challenges will be crucial in improving the HRQoL of individuals living with type 2 diabetes in Pakistan.
